# HE4 tumor marker concentration in neoplastic peritoneal effusion and in peritoneal fluid associated with benign gynecological diseases

**DOI:** 10.1186/1757-2215-7-22

**Published:** 2014-02-14

**Authors:** Anita Chudecka-Głaz, Aneta Cymbaluk-Płoska, Janusz Menkiszak, Agnieszka Sompolska-Rzechuła, Elżbieta Byra, Izabella Rzepka-Górska

**Affiliations:** 1 Department of Gynecological Surgery and Gynecological Oncology of Adults and Adolescents, Pomeranian Medical University, Al. Powstańców Wielkopolskich 72, Szczecin PL-70-111, Poland; 2 Department of Mathematics Applications in Economy of the West Pomeranian University of Technology, Janickiego 33, Szczecin 71-270, Poland; 3 Central Laboratory SPSK-2 Pomeranian Medical University, Powstańców Wlkp 72, Szczecin 70-111, Poland

**Keywords:** HE4, Tumor markers, Ascites, Ovarian cancer

## Abstract

**Background:**

The aim of our study was to evaluate the behaviour of the human epididymis protein 4 (HE4) in the peritoneal fluid encountered in various female genital diseases.

**Methods:**

We enrolled 139 patients, 40 with ovarian cancer (group I), 82 with benign diseases (group II), and 17 with other malignant neoplasms (group III). The HE4 tumor marker concentrations were determined in serum, in the peritoneal effusion and ovarian cyst/ tumor fluids, CA125 in the serum only. We compared the groups, examined correlations and determined corresponding ROC curves. We evaluated the relationship between the HE4 marker concentration in the peritoneal effusion in the group I, depending on the selected prognostic parameters.

**Results:**

The HE4 median value between the study groups did not differ statistically significantly and were as follows: in group I 3322 pmol/L, in the group II 2150 pmol/L and in the group III 627 pmol/L (p = 0.206376 for the groups I and II, p = 0.05929 for the groups I and III and p = 0.0797 for the groups II and III. In group I there were no differences found in the HE4 concentrations in the peritoneal fluid, depending on the stage, grade, the presence of neoplastic cells and the peritoneal dissemination.

**Conclusions:**

The HE4 marker concentrations in the peritoneal fluid are highly irrespective of the pathology observed in the female sexual organ. Therefore, it seems that its determinations in the peritoneal fluid are completely useless in terms of diagnostics. More research is needed on the role of the HE4 marker, especially the place of its formation and possible use in the targeted therapy.

## Introduction

The ovarian cancer is a new growth tumor whose statistics on mortality are poor despite using more and more effective and sophisticated diagnostic methods, as well as more and more novel methods of treatment. This is caused by the fact that ovarian cancer is a tumor characterized by rapid growth, by a specific peritoneal way of dissemination in an early stage of disease, which results in ascites formation [1]. Neoplastic peritoneal effusion is research material of high importance. It is associated with searching for the diagnostic usefulness of peritoneal effusion as well as with the desire to acquire detailed knowledge of all its physiopathological aspects, which could give rise to increasingly popular and more specific methods of intraperitoneal treatment [2-4].

There are many works on the behaviour of the markers, cytokines, and other substances in peritoneal effusion [5-8]. However, so far only one publication has appeared, devoted, to a negligible extent, to the research on the human epididymis protein 4 (HE4) marker in the neoplastic ovarian peritoneal effusion [9]. The HE4 overexpression in the ovarian cancer cells was demonstrated in the late nineties [10], and in 2003 in the Hellstrom studies [11] it was demonstrated as a tumor marker for ovarian cancer. At present, the HE4 glycoprotein has a proven diagnostic value [12-15], as well as prognostic significance [16-19]. However, its exact biological significance, or its possible involvement in the carcinogenesis process of ovarian cancer is not yet known.

The aim of our study was to evaluate the behaviour of the HE4 glycoprotein in the peritoneal effusion of patients with ovarian cancer and in other gynecological and non-gynecological diseases, as well as to assess its diagnostic usefulness.

## Materials and methods

139 women treated at the specialist cancer centre in 2012–2013 were enrolled in the study. The patients at the time of reporting to the Department signed informed consent for the study within the research project approved by the Commission of Bioethics of the Pomeranian Medical University. Patients who had indications for surgery were qualified as eligible for the study: i.e. patients with suspected ovarian cancer, other non-ovarian neoplasms, and patients with potentially benign gynecological diseases. The day before the scheduled surgery blood samples were taken and the CA125 and HE4 concentrations determined. Finally, those patients were qualified for the study, in whom during surgical treatment the peritoneal effusion and/or fluid from the ovarian tumor/cyst was available. Examination of the HE4 marker was carried out on the date of the fluid sample collection without having to freeze the collected material.

Following the outcome of the histopathological examination, the patients were divided into 3 groups (Table 1):

1. Group I - patients with ovarian cancer, n = 40

2. Group II-patients with gynecological and non-gynecological benign diseases, n = 82

3. Group III-patients with other malignant neoplasms, n = 17

**Table 1:** Patients characteristics

**Study group**	**Mean age**	**Number of patients**
Epithelial ovarian cancer patients	62,35	40
○ Serous	63,9	31
○ Endometrioid	60,66	6
○ Mucinous	49,66	3
FIGO I, II	53.3	10
FIGO III, IV	65.37	30
Grading 1	52	7
Grading 2	63.4	9
Grading 3	61.96	24
Other gynecological and non- gynecological conditions	41,98	82
○ Functional cysts	45,14	21
○ Ovarian endometrioma	35,71	14
○ Mature teratoma	28	9
○ Borderline epithelial tumors	41,78	9
○ Benign epithelial and gonadal tumors	50,69	16
○ Myoma	42,4	5
○ Inflammation	37,5	4
○ Cirrhosis	47,75	4
Other malignant neoplasms	56,24	17
○ Lymphoma	41	1
○ Malignant GCT	65,5	2
○ Breast cancer	56	4
○ Cervical cancer	43,6	3
○ Endometrial cancer	61,3	3
○ Stomach cancer	61,5	4

The median HE4 marker concentration in the peritoneal fluid and in the serum as well as that from serum CA125 were compared between the groups and subgroups. Additionally, the correlations between the tested markers in the serum and in the tested fluids were examined. In the ovarian cancer group the values of the studied markers were also analysed and compared, depending on the selected prognostic factors: stage, grade, presence of neoplastic peritoneal dissemination and presence or absence of neoplastic cells in the fluid. The diagnostic usefulness of the HE4 determinations in the fluid and serum, and of CA125 in serum was evaluated by determining the ROC curves. In a small number of patients the behaviour of HE4 in the fluid from the ovarian tumor/cyst was examined.

### Marker analysis

Assays were performed at the Central Laboratory of the Independent Public Hospital.

CA125 was determined with the Architect i2000 assay from Abbott Diagnostics. The normal range was 1–35 U/ml.

The serum, peritoneal and cyst/tumor fluid HE4 concentrations were measured with the Elecsys ECLIA assay from Roche running on the cobas e 601 analyser. The measurement range was 15.0-1500 pmol/L. Samples exceeding the upper range were diluted with Elecsys Diluent Multiassay. The manufacturer’s instructions were followed and control samples were within the normal range. The normal upper limit range for serum was below 70 pmol/L.

### Statistical analysis

#### The statistical analysis was performed using STATISTICA 9.1 PL program

The entire group was analysed to check the normal distribution using the Shapiro-Wilk test. Due to the fact that the data did not have normal distribution, the non-parametric U Mann–Whitney test was used to compare median values between the groups and determined confidence intervals. The scatter plots of the HE4 marker empirical points in serum, in peritoneal fluid, and in the fluid from the cyst/tumor as well as for CA125 in serum for the analysed groups I, II and III were created.

Because of the poor legibility of the scatter plots of the actual values, which is related to the fact that these characteristics do not have normal distribution, values transformation was used by means of radix 2 (log2) logarithm. The resulting diagrams allowed the clear representation of the logarithm values scatter.

In order to determine the strength of the relationship between the analysed markers the Spearman’s rank correlation coefficient was used. Selected results of this analysis were presented in the form of diagrams.

The receiver operating characteristic (ROC) curves were obtained and the area under curve (AUC) was calculated with 95% confidence intervals according to the nonparametric method of DeLong [20]. We also used this method to compare the AUCs. The level of significance was taken as *p* < 0.05.

## Results

### Comparisons of the groups and subgroups

**Figure 1:**
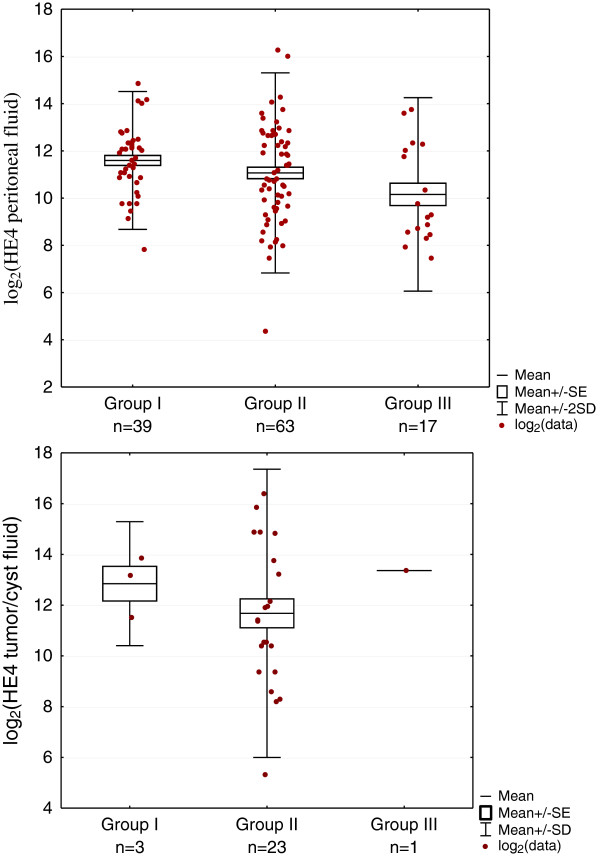
Scatterplot of HE4 concentrations in peritoneal fluid and tumor/cyst fluid in groups I (ovarian cancer), II (gynaecological and non gynaecological benign diseases) and III (other malignant neoplasms).

**Figure 2:**
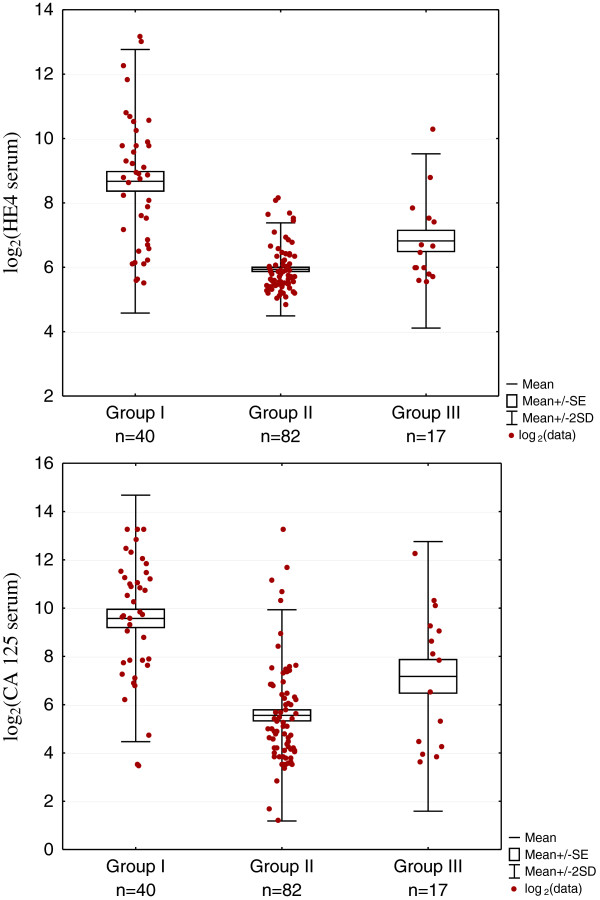
Scatterplot of serum HE4 and serum CA 125 concentrations in groups I (ovarian cancer), II (gynaecological and non gynaecological benign diseases) and III (other malignant neoplasms).

The patients characteristics are shown in Table 1. The serum marker determination was carried out in 139 patients. The peritoneal effusion was collected from 119 patients. The fluid samples from the ovarian tumor /cyst were collected from 27 women. Median HE4 values in the peritoneal fluid, in the tumor/cyst fluid and serum and median serum CA125 values are presented in Table 2 and the logarithmized values of markers are shown on Figures 1 and 2. No statistically significant differences were found for HE4 marker in the peritoneal fluid between the groups. No comparison was made between the groups concerning HE4 in tumor/cyst fluids due to small number of cases.

**Table 2:** HE4 concentrations in serum, peritoneal fluid and tumor/cyst fluid and CA125 concentrations in serum in examined groups

	**HE4-peritoneal fluid [pmol/L]**	**HE4- serum [pmol/L]**	**CA125- serum [mIU/ml]**	**HE4 –tumor/cyst fluid [pmol/L]**
Group I	3322	452.05	885.45	9162
[median/95% CI]	[10.78-6633.22]	[-272.37-1176.47]	[-732.04-2502.94]	[9157.69-9166.3]
Group II	2150	53.25	33.250	2694
[median/95% CI]	[-1334.88-5634.88]	[28.46-78.04]	[-8.01-74.51]	[-1902.9-7290.9]
p value	0.206376	0.0000001	0.0000001	-
Group I	3322	452.05	885.45	9162
[median/95% CI]	[10.78-6633.22]	[-272-1176.47]	[-732.04-2502.94]	[9157.69-9166.3]
Group III	627.4	87.6	231.6	10522
[median/95% CI]	[-132.1-1386.9]	[17.63-157.57]	[-191.76-654.96]	-
p value	0.05929	0.001652	0.010584	-
Group II	2150	53.25	33.250	2694
[median/95% CI]	[-1334.88-5634.88]	[28.46-78.04]	[-8.01-74.51]	[-1902.9-7290.9]
Group III	627.4	87.6	231.6	10522
[median/95% CI]	[-132.1-1386.9]	[17.63-157.57]	[-191.76-654.96]	-
p value	0.0797	0.003943	0.043166	-

There were no statistical differences found in group I, depending on the histopathological type of the HE4 values in the peritoneal fluid and serum. Due to the small number of cases of mucinous and endometrioid carcinomas the statistical analysis was only performed between the subgroup serous vs mucinous + endometrioid, p = 0.4532 (for the peritoneal fluid) and p = 0.2499 (for the blood serum). The following median values and 95% confidence interval of HE4 in peritoneal fluid were found in serous 4087 (60.18-8113.82) pmol/L, endometrioid 3664 (-2128.47-5817.67) pmol/L and mucinous carcinomas 1844.6 (-323.65-8683.65) pmol /L .

The median values of HE4 in the peritoneal fluids, the tumor/cyst fluids and in serum of CA125 and HE4 in the subgroups of the group II are presented in Table 3. In inflammatory conditions, myomas and for cirrhosis, only peritoneal fluid and serum were analysed. No median values were compared within the subgroup of myomas, hepatic cirrhosis and inflammations due to the small number of cases. In other subgroups of the group II the significant differences of HE4 in the peritoneal fluid were found between the functional cysts and teratomas, (p = 0.0391), borderline malignancy tumors and teratomas, (p = 0.008), myomas and teratomas (p = 0.0225) and between the endometrial cysts and teratomas, (p = 0.0217).

**Table 3:** HE4 concentrations in serum, peritoneal fluid and tumor/cyst fluid and CA125 concentrations in serum in examined subgroups of group II

	**HE4-peritoneal fluid [pmol/L]**	**HE4- serum [pmol/L]**	**CA125- serum [mIU/ml]**	**HE4 –tumor/cyst fluid [pmol/L]**
Functional cysts	1799	52.6	17.3	2097
[median/95% CI]	[-1152.67-4750.66]	[19.53-85.67]	[5.54-29.07]	[-1208.38-5402.38]
Endometrial cysts	5248	45.05	56.6	15674
[median/95% CI]	[-2563.74-13059.74]	[31.66-58.44]	[7.89-105.32]	[-27785.5-59133.53]
Mature teratoma	405.3	47.65	30.15	384.3
[median/95% CI]	[3.13-807.47]	[30.86-64.44]	[0.59-59.71]	[-342.2-1110.8]
Borderline tumors	4772	84.3	39.6	30000
[median/95% CI]	[-2551.87-12095.87]	[30.34-138.26]	[-8.83-88.03]	-
Benign epithelial	2150	56.850	24.4	3872
/gonadal tumors	[-5878.85-10178.85]	[43.64-70.07]	[-23.97-72.76]	[-29543.1-37287.05]
[median/95% CI]				
Myomas	4822	58.3	90.1	-
[median/95% CI]	[-2435-12079.64]	[36.79-79.81]	[-841.26-1021.46]	-
Inflammation	498.6	65.7	113.25	-
[median/95% CI]	[-573.25-1570-45]	[57.09-74.31]	[-136.41-362.91]	-
Cirrhosis	675.95	112.6	743.75	-
[median/95% CI]	[-113.78-1465.68]	[28.17-196.83]	[-998-2458.5]	-

### Linear correlations

**Figure 3:**
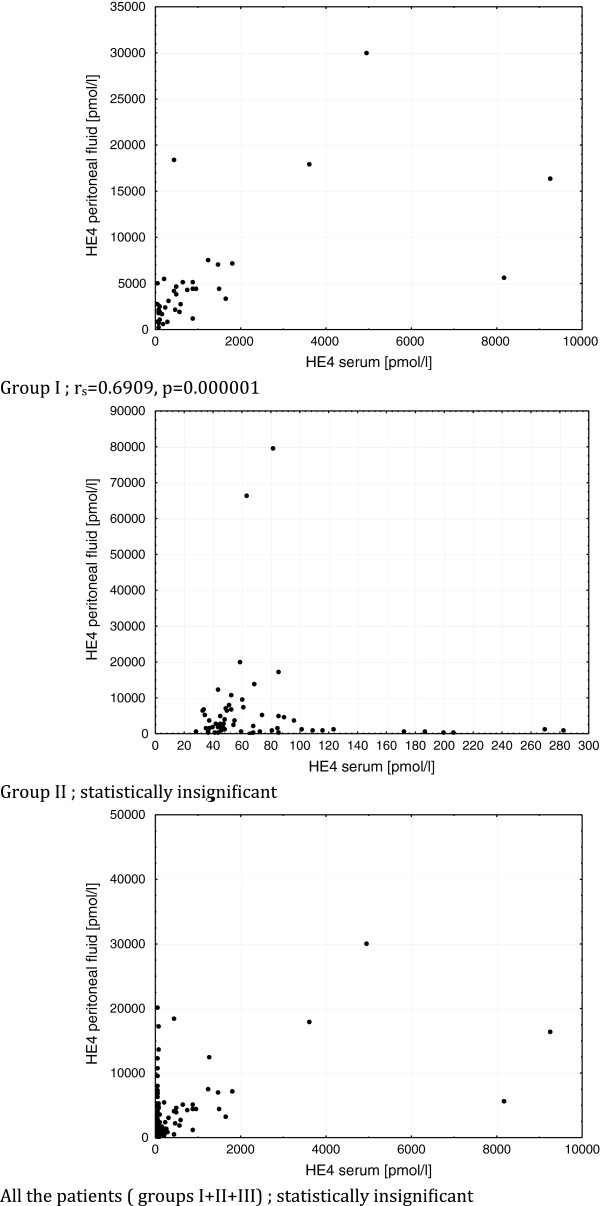
Spearman’s correlations between the HE4 concentration in serum and the HE4 concentration in the peritoneal fluid in group I, II, and in all examined patients.

Assessment was made, whether there is a relationship between the tested markers using the Spearman’s rank correlation coefficient. No significant correlation was found between HE4 in serum and HE4 in the peritoneal fluid; the HE4 serum versus the HE4 tumor/cyst fluid; the CA125 serum and the HE4 tumor/cyst fluid and the CA125 serum versus HE4 in peritoneal fluid when conducting the analysis of the total studied patient. The only positive significant relationship was found between serum levels of CA125 and serum levels of HE4 (r_s_ = 0.6678, p = 0.000001). When analysing the particular groups, there was a statistically significant, strong correlation between the HE4 marker concentration in the serum and its concentration in the peritoneal fluid in ovarian cancers group (r_s_ = 0.6909, p < 0.000001), Figure 3. In the groups II and III no correlation was observed.

### Tested markers depending on the selected prognostic factors

There were no statistically significant differences found in the HE4 concentrations in the peritoneal fluid, depending on the stage, grade, peritoneal dissemination and presence or absence of neoplastic cells. The HE4 serum values differed significantly between low-advanced (FIGO I and II) and high-advanced (FIGO III and IV) stages, high- and low-differentiated tumors. There is no significant difference depending on presence or absence of peritoneal dissemination (Table 4). The median CA125 concentrations in serum manifested a statistically significant difference only depending on the grade (Table 5).

**Table 4:** HE4 concentrations in serum and peritoneal fluid, CA125 concentrations in serum in patients with ovarian cancer (group I) according to FIGO stage, peritoneal carcinomatosis and presence of neoplastic cells in peritoneal fluid

	**HE4-peritoneal fluid [pmol/L]**	**HE4- serum [pmol/L]**	**CA125- serum [mIU/ml]**
FIGO I, II	2306	80.45	573.55
[median/95% CI]	[-945.89-5558.49]	[9.64-151.26]	[-680.59-1827.69]
FIGO III, IV	4298	576.55	1288.45
[median/95% CI]	[-67.16-8663.16]	[-185.61-1338.71]	[-923.67-3500.57]
p value	0.1043	0.0001	0.0975
Peritoneal carcinomatosis	4298	576.55	1288.45
YES	[-67.16-8663.16]	[-185.61-1338.71]	[-923.67-3500.57]
[median/95% CI]			
Peritoneal carcinomatosis	2306	80.45	573.55
NO	[-945.89-5558.49]	[9.64-151.26]	[-680.59-1827.69]
[median/95% CI]			
p value	0.1043	0.0001	0.0975
Neoplastic cells in	4412	555.6	826.5
Peritoneal fluid YES	[-211.66-9035.66]	[-239.48-1350.68]	[-751-2404]
[median/95% CI]			
Neoplastic cells in	3236	391.5	1085.75
Peritoneal fluid NO	[353.69-6116.31]	[-268.25-1051.25]	[-1153.45-3324.94]
[median/95% CI]			
p value	0.3868	0.2381	1.000

**Table 5:** HE4 concentrations in serum and in peritoneal fluid, CA125 concentrations in serum, in patients with ovarian cancer (group I) according to cancer grade

	**HE4-peritoneal fluid [pmol/L]**	**HE4- serum [pmol/L]**	**CA125- serum [mIU/ml]**
Grade 1	1844.6	49.9	75
[median/95% CI]	[-844.67-4533.87]	[37.41-62.39]	[-58.27-208.27]
Grade 2	4180	439.3	919.5
[median/95% CI]	[-28.69-8388.69]	[-452.11-1330.71]	[-845.7-2684.7]
p value	0.1044	0.0093	0.0137
Grade 1	1844.6	49.9	75
[median/95% CI]	[-844.67-4533.87]	[37.41-62.39]	[-58.27-208.27]
Grade 3	4444	548.35	1770.05
[median/95% CI]	[263.07-8624.93]	[-235.46-1332.16]	[-757.23-4297.33]
p value	0.1337	0.0011	0.0019
Grade 2	4180	439.3	919.5
[median/95% CI]	[-28.69-8388.69]	[-452.11-1330.71]	[-845.7-2684.7]
Grade 3	4444	548.35	1770.05
[median/95% CI]	[263.07-8624.93]	[-235.46-1332.16]	[-757.23-4297.33]
p value	0.9608	0.7587	0.8154
Grade 1 and 2	2958	97.2	215.6
[median/95% CI]	[48.29-5867.7]	[-13.51-207.91]	[-204.63-635.83]]
Grade 3	4444	548.35	1770.05
[median/95% CI]	[263.07-8624.93]	[-235.46-1332.16]	[-757.23-4297.33]
p value	0.4141	0.0331	0.0375

### ROC curves

**Figure 4:**
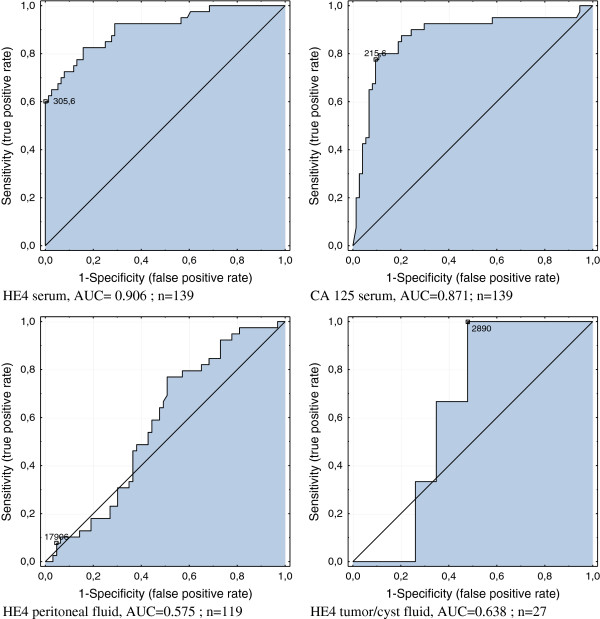
ROC curves for HE4 in serum, in peritoneal fluid and in tumor/cyst fluids and CA125 in serum of examined patients.

The ROC curves were analysed for the investigated tumor markers and the areas under the curve (AUC) were compared. HE4 marker in serum had the highest diagnostic value in this study - its AUC is 0.905, and CA125 AUC is 0.871. The AUC value for HE4 in the peritoneal fluid and in the fluid from the tumor/cyst was equal to 0.575 and 0.638, respectively, (Figure 4). Statistically significant differences were found between AUC of HE4 from the peritoneal fluid and serum, (p = 0.0001) and between the HE4 from peritoneal fluid and tumor fluid, (p = 0.0009).

## Discussion

In cases of neoplastic dissemination, the ascites formation mechanism is complex. It is associated with the increased tumor microvascular permeability, as well as with the fact that the tumor itself is capable of its production. The peritoneal fluid observed in the patients with ovarian cancer is the subject of considerable research in connection with great hopes for the creation of the targeted intraperitoneal therapy [2]. For this to happen it is necessary to know the exact physiopathology of that fluid.

The aim of our study was first of all to examine the behavior of the HE4 marker concentrations in the peritoneal fluid of different origin, in the course of both neoplastic diseases and benign illness.

The HE4 tumor marker has already been approved for the diagnosis and monitoring of ovarian cancer [11-14]. We also know more and more about its prognostic significance [16-19]. However, what has so far been established in terms of its biological function in physiological conditions and in ovarian cancer? A hypothesis was advanced that the WFDC2 protein may be a component of the innate immune response in the lung, nose and oral cavity [21,22]. By immunohistochemical examination the expression of HE4 was found in a normal epithelium of the genital tract in women, i.e. endocervical, endometrial glands, fimbriae of fallopian tubes and in Bartholin's glands. In neoplastic tumors the greatest expression was found in serous ovarian cancer with varying grades of malignancy (including borderline tumors), and the majority of endometrial and clear cell type. No expression was found in the germ cell, gonadal tumors and mucinous carcinomas [23]. Drapkin et al. [24] also demonstrated the expression of WFDC2 glycoprotein in inclusion ovarian cysts of the Mullerian origin ovarian cancer, pointing at possible involvement of these lesions in the process of ovarian carcinogenesis. The lack of changes in the HE4 concentrations in serum in the course of a woman’s menstrual cycle indicates a lack of correlation with and dependence on hormones of the menstrual cycle [25,26]. Data on the role of HE4 in the carcinogenesis are inconsistent. Gao et al. [27] demonstrated that the transfer of “exogenous HE4 gene” to the ovarian cancer cell lines significantly promotes cell apoptosis and it can contribute to the protective role of this gene in the ovarian cancer progression process. Quite different are the conclusions of the study conducted by Zou et al. [28], who investigated nine cell lines. They found out that the HE4 gene silencing results in cell division stopping in the G0/G1 phase which in turn is associated with the inhibition of proliferation, migration and invasion of the ovarian cancer cells. The HE4 participation in promoting the neoplastic tumor growth was also demonstrated by other authors [29,30]. Lu et al. [30] demonstrated that the HE4 expression in cancer cells is associated with greater adhesion, migration and proliferation which may be dependent on the EGFR-MAPK cascade. HE4 protein, in the opinion of the authors, should be considered in the aspects of targeted therapy.

Until now two papers have been published in which the HE4 marker concentration was examined in body fluids apart from blood [9,31]. Eisammak et al. [31] assessed HE4 concentrations in the transudative and exudative pleural fluid of different origins. They found statistically higher HE4 concentrations in the exudative neoplastic fluid (2878.5 pmol/L) compared to the pleural fluid in benign lung diseases (transudate-305 pmol/L, exudate-358.3 pmol/L). In addition, HE4 serum levels correlated with the HE4 concentrations in the exudative fluid. The area under the curve was even greater for HE4 in the pleural fluid qualified as a good diagnostic marker. The results presented by Braicu et al. [9] are most similar to the results of our research because they examined HE4 concentrations in neoplastic peritoneal fluid. But results obtained in their study did not refer to diagnostic value of HE4 and because of that the author did not examine ascites in benign gynaecological conditions. The mean value of HE4 in ovarian neoplastic peritoneal fluid was equal to 4339 pmol/L (2117–7752 pmol/L), and in our study, we obtained similar results of HE4 in peritoneal neoplastic effusion (mean 5024.19 pmol/L, range: 223–30000 pmol/L; median 3322 pmol/L, 95% CI 10.78-6633.22). Similarly to our study, the authors found no differences in mean values of HE4 in the peritoneal fluid depending on the stage of ovarian cancer, and its differentiation. The authors [9] showed that the HE4 concentration is dependent on the amount of peritoneal fluid: the greater the amount of the fluid, the greater concentrations were observed by the authors. In our research we did not divide the group of tumors according to their amount of peritoneal fluid, but in small liquid amounts we detected quite often very high HE4 concentrations, also in the benign diseases. Sometimes the HE4 values exceed the concentrations found in malignant ascites. According to the authors [9], the HE4 marker determined in peritoneal fluid also has a prognostic significance and correlates with selected parameters of survival: with OS in univariate analysis (p = 0.044) and platinum response (p = 0.023).

The results of our study are somewhat surprising. For the first time we have examined HE4 concentrations in the peritoneal ovarian neoplastic fluid, also in a variety of benign diseases and other cancers, not only gynecological ones. Although the median values differed between the groups, we did not find any statistically significant difference between the investigated populations. The HE4 concentrations in the peritoneal fluid did not differ between ascites in the course of the ovarian cancer, ascites of other new growth tumors or in comparison with benign pathological states such as uterine myomas, benign ovarian cysts or inflammatory states, accompanied by the presence of the peritoneal fluid. At this point a question arises, whether HE4 tested in the peritoneal fluid is definitely produced by the new growth tumor cells, as was suggested by other authors. It appears that the high HE4 values observed in the peritoneal fluid in case of benign diseases are inconsistent with these established facts. The higher HE4 concentrations in the peritoneal fluid, accompanied by peritoneal dissemination or where cancer cells were present in the fluid, are also statistically insignificant. The maximum HE4 values, both in the peritoneal fluid and in the fluids from the tumor/cyst were found in the patients from group II but not in the ovarian cancer patients, and they were as follows: 79610 pmol/L in the peritoneal fluid of patients with mucinous cystadenoma and 86780 pmol/L in the fluid of the hydrosalpinx, respectively the maximum values of HE4 in ascites observed in the course of ovarian cancer were only 30 000 pmol/L, and in the liquid from a malignant epithelial tumor only 15 068 pmol/L. We found no correlation, neither, between the HE4 values in serum and peritoneal fluid in the whole studied population and only in the group of women with ovarian cancer, which may indicate increased vascular permeability of the tumor. Based on our research, the HE4 determinations in the peritoneal fluid cannot be considered a valuable diagnostic method (AUC = 0.575).

In the summary of our results, it appears that the HE4 marker high concentrations in the peritoneal fluid are not specific to ovarian cancer patients. Most probably, the determination of HE4 in the peritoneal fluid as well as in the fluid from ovarian cyst/tumor has no diagnostic value. More research is needed which shall be aimed at the assessment of HE4 formation whose high concentrations are detectable in most pelvic diseases.

## Competing interests

The authors declare that they have no competing interests.

## Authors’ contributions

AC-G have made substantial contributions to conception and design, planned and ran the experiments, collected data, performed analysis and interpretation of the results, review the literature and wrote the manuscript. AC-P have contributed in collected data. JM have contributed in literature review. EB supervised laboratory diagnostics. AS-R supervised statistical analysis. IR-G have given final approval of the version to be published. All authors read and approved the final manuscript.
